# HPV, EBV, CMV, and HSV in Head and Neck Cancer: Molecular Detection, Seroprevalence, and Clinical Correlations

**DOI:** 10.3390/biology14111523

**Published:** 2025-10-30

**Authors:** Mustafa Onel, Hayriye Kirkoyun Uysal, Murat Ulusan, Utkucan Ayeser, Kutay Sarsar, Yasemin Ayse Ucar, Ozlem Yoldas, Arat Hulikyan, Fulya Gurkan Kiraz, Ali Mert Uysal, Mehmet Celik, Mehmet Demirci, Ali Agacfidan

**Affiliations:** 1 Department of Medical Microbiology, Istanbul Medical Faculty, Istanbul University, Fatih 34093, Istanbul, Turkey; onelm@istanbul.edu.tr (M.O.); kutay.sarsar@istanbul.edu.tr (K.S.); arathulikyan@hotmail.com (A.H.); aagacfidan@hotmail.com (A.A.); 2 Department of Otolaryngology–Head and Neck Surgery, Istanbul Medical Faculty, Istanbul University, Fatih 34093, Istanbul, Turkey; mulusan@istanbul.edu.tr (M.U.); utkucan.ayeser@istanbul.edu.tr (U.A.); mehmetcelik@istanbul.edu.tr (M.C.); 3 Department of Medical Microbiology, Faculty of Medicine, Istanbul Beykent University, Büyükçekmece 34500, Istanbul, Turkey; ayseucar@beykent.edu.tr; 4 Vocational School of Health Services, Altınbaş University, Bakırköy 34147, Istanbul, Turkey; ozlem.yoldas@altinbas.edu.tr; 5 Department of Medical Microbiology, Institute of Health Sciences, Istanbul University, Fatih 34126, Istanbul, Turkey; fulya.gurkan@ogr.iu.edu.tr; 6 Medical Faculty, Acıbadem University, Ataşehir 34638, Istanbul, Turkey; ali.uysal@live.acibadem.edu.tr; 7 Department of Medical Microbiology, Faculty of Medicine, Kirklareli University, Kırklareli 39100, Kırklareli, Turkey; mdemirci1979@gmail.com

**Keywords:** head and neck cancer, viral STD agents, oncogenic viruses

## Abstract

(1) Background: This study investigated the presence of human papillomavirus (HPV), HPV genotypes, Epstein–Barr virus (EBV), cytomegalovirus (CMV) and herpes simplex virus (HSV) in patients with Head and Neck Cancer (HNC) at both molecular and serological levels. (2) Methods Fifty patients with histopathologically confirmed HNC who were admitted to the Department of Otorhinolaryngology, Istanbul Faculty of Medicine. Viral DNA was detected using quantitative real-time PCR, and serological IgM and IgG antibodies were analyzed using the CMIA method; (3) Results: In blood samples, CMV and HSV DNA were not detected, whereas EBV DNA was identified in 2% and HPV DNA in 4% of patients. In tumor tissues, CMV DNA was detected in 8%, EBV DNA in 10%, and HPV DNA in 6%; HSV DNA is 6%. HPV genotypes 18, 45, and 69 were found in tissue samples. Serologically, IgG positivity for CMV, EBV, and HSV-1 exceeded 90%, whereas IgM positivity was low and not statistically significant; (4) Conclusions: HPV, EBV, and CMV DNA were detected at low frequencies in patients with HNC, while HSV DNA was absent. These findings underline the need for larger multi-center studies and support the consideration of routine viral screening, particularly for HPV, in specific tumor subtypes.

## Simple Summary

Head and neck cancers constitute a global health burden, and viral pathogens have been implicated in their etiopathogenesis. This study specifically examined cytomegalovirus, Epstein–Barr virus, human papillomavirus and herpes simplex virus at molecular and serological levels in fifty patients in total with histopathologically confirmed head and neck cancer treated at the Department of Otorhinolaryngology, Istanbul Faculty of Medicine. Blood and tumor tissue specimens were collected at diagnosis and analyzed using quantitative molecular assays and serological tests for antibody responses. In peripheral blood, cytomegalovirus and herpes simplex virus DNA were not detected, while Epstein–Barr virus DNA was identified in one patient and human papillomavirus DNA in two. In tumor tissue, cytomegalovirus DNA was detected in five patients, Epstein–Barr virus DNA in six, and human papillomavirus DNA in three, whereas herpes simplex virus DNA was absent. Genotyping revealed human papillomavirus types 18, 45 and 69 in tumor specimens, with one patient positive for type 18 in both blood and tissue. Serology demonstrated that over ninety percent of patients had immunoglobulin G reactivity indicative of past exposure, whereas immunoglobulin M responses were uncommon. Overall viral detection rates were low, yet findings support further multicenter studies and consideration of targeted screening and vaccination strategies.

## 1. Introduction

Head and neck cancers constitute a significant public health concern worldwide due to their high morbidity and mortality [1]. While environmental factors, such as tobacco and alcohol use particularly in squamous cell carcinomas (SCCs) remain the main etiological contributors, recent studies indicate that viral infections may also play a meaningful role in tumor pathogenesis [2]. The primary viral agents investigated in association with head and neck malignancies include human papillomavirus (HPV), Epstein–Barr virus (EBV), cytomegalovirus (CMV), and herpes simplex virus (HSV) [2,3,4,5,6]. The aim of this study was to investigate the prevalence and distribution of CMV, EBV, HPV, and HSV in HNC patients at both molecular and serological levels and to explore their potential epidemiological and clinical significance, contributing to future discussions on the feasibility of targeted viral screening and vaccination strategies in this population.

Extensive studies and meta-analyses have demonstrated the oncogenic role of HPV, particularly in specific subtypes such as oropharyngeal and oral cavity SCCs [3,4]. EBV is almost universally associated with nasopharyngeal carcinoma, while its prevalence in other head and neck tumors varies depending on geographic and ethnic factors [5,6]. CMV and HSV are not primarily linked to direct oncogenic activity but are thought to influence the tumor microenvironment and modulate immune responses [7,8]. Nevertheless, the overall prevalence of these viruses in HNCs, their associations with clinical and histopathological features, and their potential impact on tumor behavior remain limited and controversial [9].

Currently, both molecular techniques, such as PCR-based viral DNA analysis, and serological assays, including IgM and IgG antibody measurements, are used to detect viral agents in HNCs. However, the clinical relevance of viral DNA positivity and seroprevalence—particularly considering regional epidemiological differences and tumor subtypes—has not been fully established [10]. Furthermore, most studies originate from Western countries or regions with high incidence rates, and comprehensive screenings that include multiple viral agents are still scarce in transitional regions such as Turkey.

Viral infections, particularly those caused by HPV, EBV, CMV, and HSV, have been implicated in the pathogenesis of HNCs; however, their prevalence, distribution across tumor subtypes, and clinical significance remain incompletely understood. The aim of this study is to evaluate the presence of HPV, including its genotypes, as well as EBV, CMV, and HSV at both molecular and serological levels, using blood and tumor biopsy samples from patients with HNC admitted to the Department of Otorhinolaryngology, Istanbul Faculty of Medicine, and to assess their associations with demographic and clinical factors, providing insight into their potential role in tumor development and regional epidemiological patterns.

## 2. Materials and Methods

### 2.1. Study Design and Participants

This study was conducted by including 50 patients who were admitted to the Otorhinolaryngology Outpatient Clinic of Istanbul Faculty of Medicine between April 2023 and April 2024 and were histopathologically diagnosed with HNC. The study was approved by the Clinical Research Ethics Committee of Istanbul University Faculty of Medicine on 7 November 2023 (Approval No: 2234909). The age, sex, tumor localization, clinical stage, and risk factors (smoking, alcohol consumption, comorbidities) of the included patients were recorded. Venous blood samples and tumor tissue biopsy tissues were collected from all patients.

For molecular analyses, blood samples were collected in EDTA-containing anticoagulant tubes (Vacusera, Disera Ltd., Izmir, Turkey), while tissue biopsy samples were obtained in physiological saline under sterile conditions. For serological analyses, blood samples were drawn into gel-containing, anticoagulant-free tubes (Vacusera, Disera Ltd., Izmir, Turkey), and serum was obtained by centrifugation.

Patients who had previously received antiviral treatment for any viral infection, had immunodeficiency, systemic infections, or hematological malignancies were excluded from the study. Clinical and demographic data were obtained from the hospital information system and patient records.

All patients provided written informed consent before participating in the study. The procedures followed were in accordance with the ethical standards of the committee responsible and the Helsinki Declaration.

### 2.2. Viral DNA Analysis—Quantitative Real-Time PCR (qPCR)

Nucleic acid extraction was performed using a commercial viral DNA isolation kit (MinElute^®^ Virus Spin Kit, Qiagen, Hilden, Germany) according to the manufacturer’s protocol. The extracted DNA samples were subjected to qPCR analysis using primers and probes specific to each virus. Positive and negative control samples were included in each run. Results were interpreted based on Ct (Cycle threshold) values, and positivity was determined using the threshold values recommended by the manufacturer. Each sample was analyzed under double-blind conditions by at least two experienced researchers.

The presence of viral DNA for CMV, EBV, HPV, and HSV was investigated in both venous blood samples and tissue biopsy tissues using quantitative real-time polymerase chain reaction (qPCR), with a specific kit employed for each pathogen: HSV QLP 2.1 Real-Time PCR Kit, Fluorion HPV Screening QNS 1.1 Real-Time PCR Kit, Fluorion CMV QNP 3.0, and Epstein–Barr Virus QNP 1.0 Real-Time PCR Kit (IONTEK Pharmaceuticals, Diagnostics and Biotechnology R&D Corp., Istanbul, Turkey). The geneMAP™ HPV 29 Genotyping Kit (Genmark, Istanbul, Turkey) was used for genotyping of 29 High Risk and Low Risk Human Papilloma Viruses (16, 18, 26, 31, 33, 35, 39, 40, 42, 43, 44, 45, 51, 52, 53, 54, 56, 58, 59, 61, 66, 68, 69, 70, 73, 81/82, 6/11) via qPCR according to manufacturer’s instruction

### 2.3. Serological Tests

IgM and IgG levels for CMV, EBV, HSV-1/2, and HPV were analyzed using the Enzyme-Linked Immunosorbent Assay (ELISA) method. The analyses were performed using kits and protocols recommended by the manufacturer, and the thresholds for positivity and negativity were determined based on the in-kit validation values. All samples were thawed only once prior to analysis and were not subjected to repeated freeze–thaw cycles. Results were reported both quantitatively and as positive/negative according to the manufacturer’s cut-off values.

#### 2.3.1. HPV IgM and IgG Tests

A commercial kit based on the capture ELISA principle was used to measure HPV IgM and IgG levels (Human Papillomavirus Antibody, HPV-IgG ELISA Kit, BT Lab, Shanghai Korain Biotech Co., Shanghai, China). According to the kit’s evaluation criteria, samples with a signal-to-cutoff ratio (S/Co) < 1.0 were considered negative, and those with S/Co > 1.2 were considered positive for HPV IgM, based on optical density measurements at 450 nm wavelength. For HPV IgG, samples with levels < 0.5 IU/mL were considered negative, and those > 0.5 IU/mL were considered positive. Due to tests instructions for IgM CV (%) = SD/mean × 100 Intra-assay: CV < 10% Inter-assay: CV < 12%. For IgG CV (%) = SD/mean × 100 Intra-assay: CV < 10% Inter-assay: CV < 12%.

#### 2.3.2. EBV IgM and IgG Tests

A commercial kit based on the capture ELISA principle was used to measure EBV IgM (targeting Epstein–Barr Virus Nuclear Antigen—EBNA) and IgG (targeting Viral Capsid Antigen—VCA) levels (DIA PRO, Milan, Italy). According to the kit’s evaluation criteria, for EBV EBNA-IgM, samples with a signal-to-cutoff ratio (S/Co) < 1.0 were considered negative, and those with S/Co > 1.2 were considered positive, based on optical density measurements at 450 nm wavelength. For EBV VCA-IgG, samples with levels < 0.5 arbU/mL were considered negative, and those > 0.5 arbU/mL were considered positive. EBV IgG sensitivity and specifity are >98%. EBV IgM sensitivity and specifity are ≥98%.

#### 2.3.3. CMV IgM and IgG Tests

A commercial kit based on the capture ELISA principle was used to measure CMV IgM and IgG levels (DIA PRO, Milan, Italy). For CMV IgM, according to the kit’s evaluation criteria, samples with a signal-to-cutoff ratio (S/Co) < 1.0 were considered negative, while those with an S/Co > 1.2 were considered positive, based on optical density measurements at 450 nm wavelength. For CMV IgG, samples with levels < 0.5 IU/mL were considered negative, and those > 0.5 IU/mL were considered positive. CMV IgM and CMV IgG sensitivity and specifity are >98%.

#### 2.3.4. HSV 1/2 IgM and IgG Tests

A commercial kit based on the capture ELISA principle was used to measure HSV-1/2 IgM and IgG levels (DIA PRO, Milan, Italy). According to the kit’s evaluation criteria, samples with a signal-to-cutoff ratio (S/Co) < 1.0 were considered negative, and those with S/Co > 1.2 were considered positive for HSV-1/2 IgM, based on optical density measurements at 450 nm wavelength. For HSV-1/2 IgG, samples with levels < 0.5 arbU/mL were considered negative, and those > 0.5 arbU/mL were considered positive. HSV IgM and HSV IgG sensitivity and specifity are >98%.

### 2.4. Statistical Analysis

All statistical analyses were performed using SPSS version 26.0 for Windows (IBM Corporation, Armonk, NY, USA). Statistical analyses were performed to evaluate associations between patient characteristics (age, gender, tumor localization, histopathological diagnosis) and viral parameters (DNA and antibody positivity for HPV, EBV, CMV, and HSV) using Chi-square, Fisher’s exact, Mann–Whitney U, or Kruskal–Wallis tests, as appropriate. A *p*-value of <0.05 was considered statistically significant.

## 3. Results

In this study, a comprehensive evaluation was conducted on demographic characteristics, tumor distribution, lifestyle factors, molecular-level viral DNA positivity and serological markers based on data obtained from 50 patients diagnosed with HNC. The findings were assessed in relation to both blood and tissue viral loads as well as clinical variables such as age, sex, and tumor subtypes.

The mean age of participants was 57.9 ± 13.0 years, ranging from 24 to 86, indicating a patient population that may be influenced by age-related immunological changes. Regarding gender distribution, 73.1% of the patients were male (n = 38) and 23.1% were female (n = 12), consistent with existing literature indicating a higher prevalence of HNCs in men. The most common histopathological diagnoses were laryngeal SCC (34.6%), tongue SCC (21.2%), and retromolar SCC (7.7%). Less frequent tumor types included hypopharyngeal SCC, tonsillar SCC, and nasopharyngeal carcinoma (Table 1).

Among lifestyle factors, smoking was identified in 88% of patients (n = 44), with a high mean pack-year index, supporting its significant role in the development of non-HPV-related HNCs. Alcohol consumption was reported in only 18% (n = 9) of patients; although lower than in Western populations, it remains notable given its synergistic carcinogenic effects.

In molecular testing, CMV and HSV DNA were not detected in any blood samples. EBV DNA was detected in one patient (2%), and HPV DNA in two patients (4%), all of whom were male and between 50 and 69 years old. However, subgroup analysis revealed no statistically significant association with age or sex (*p* > 0.05). These low positivity rates may be attributed to the short viremic phase or the challenge of detecting latent infections in peripheral blood using PCR. Latent viruses like EBV and HPV may be more accurately identified through tissue-based assessments (Table 2). Comparison of blood and tissue DNA results showed that viral DNA detection in tissue samples was more frequent, suggesting that local viral persistence may not always coincide with systemic viremia.

Tissue-based DNA analysis showed higher positivity rates: CMV DNA was detected in 4 patients (8%), EBV DNA in 5 patients (10%), and HPV DNA in 3 patients (6%). HSV DNA detected in 3 patients (6%). HPV DNA positivity varied significantly by tumor subtype and was found at higher rates in tongue SCC (18.2%) and retromolar SCC (50%) cases. This distribution was statistically significant (*p* = 0.034), supporting the oncogenic role of HPV particularly in oropharyngeal tumors. By sex, CMV and EBV DNA positivity was observed only in males, while HPV DNA positivity was detected in 16.7% of females and only 2.6% of males, a difference that, although not statistically significant (*p* = 0.14), is noteworthy. One patient was HPV-positive in both blood and tissue, with genotype 18 detected in tissue. HPV genotypes 18, 45, and 69 were found in tissue samples, while genotype 18 and one undetermined type were detected in blood. 

Serological testing revealed high rates of IgG antibodies, reflecting past infections within the general population. CMV IgG (94%), EBV IgG (94%), and HSV-1 IgG (98%) positivity rates suggest widespread prior exposure to these viruses. Conversely, IgM antibodies indicative of acute infections—were detected at low rates: CMV IgM (6%), EBV IgM (2%), HSV-1 IgM (0%), HSV-2 IgM (2%), and HPV IgM (8%). This suggests a low prevalence of active primary infections in the study cohort (Table 3).

Age-stratified serological analysis revealed that IgG positivity rates approached 100% in the 50–69 age group. By gender, CMV IgG positivity was 100% in females and 92.1% in males. IgG antibodies against HPV were rare, detected in 6% of males and 0% of females. Comparisons of these serological markers by age and sex revealed no statistically significant differences (*p* > 0.05).

In summary, viral DNA positivity was low in blood samples but notably higher in tissue biopsies. Viruses such as HPV, EBV, and CMV may play a more prominent role in specific tumor subtypes, and such associations are better evaluated through tissue-based analyses. Serologically, past viral infections were found to be highly prevalent, while markers of active infection were observed at very low rates.

## 4. Discussion

While the dominant role of tobacco and alcohol in the etiology of HNCs has been well established for decades, an increasing number of studies over the past decade have highlighted the significant contribution of viral infections. Although the exact impact of viral agents on the development, progression, and treatment response of head and neck tumors remains incompletely understood, several viruses, especially HPV and EBV, have been identified as potential key factors in certain tumor subtypes [11,12]. In this study, a comprehensive multi-viral screening at both molecular (blood and tissue PCR) and serological levels revealed the associations between viral positivity and clinical/demographic variables in a regional cohort of patients with HNC. 

The significant variation in HPV DNA positivity across tumor subtypes in tissue samples (*p* = 0.005), particularly its higher detection in tongue SCC (18.2%) and retromolar SCC (50%) compared to other tumor sites such as laryngeal SCC (5.8%) and hypopharyngeal SCC (0%), suggests a central role for HPV in the pathogenesis of cancers located in these anatomical regions. This finding aligns with international data reporting HPV as a major oncogenic factor in oropharyngeal and oral cavity SCCs [13,14]. In a recent systematic review and meta-analysis, the overall prevalence of HPV-positive oropharyngeal squamous cell carcinoma (excluding tonsils and base of tongue) was found to be 20% (95% CI: 13–30%) [4]. In a study conducted by Uhlrich et al. in 2024, HPV positivity was reported in 65.8% of oropharyngeal squamous cell carcinomas [15]. We found HPV DNA positivity in tissue biopsy samples; we detected HPV 18, 45 and 69 also we found HPV 18 in blood samples. However, HPV 16 was not detected in our study. HPV 16 is typically the most prevalent genotype in head and neck squamous cell carcinomas. This absence may reflect regional genotype variation; methodological factors such as assay sensitivity or specificity. Larger, standardized studies are warranted to clarify the regional distribution and clinical relevance of HPV genotypes in HNCs. This finding further highlights the prominent role of HPV in the development of oropharyngeal cancers and underscores the importance of vaccination and early detection strategies in this field. Our findings regarding the low HPV DNA detection rate in laryngeal SCCs (5.8%) are in line with previous meta-analyses, such as Vani et al. (2024), who reported a pooled HPV DNA prevalence of 13% (95% CI: 8–19%) across laryngeal cancer studies in India, with individual studies ranging from 0% to 30% depending on detection methods [16].

Regarding EBV, the absence of a significant difference in tissue DNA positivity among tumor subtypes suggested that EBV does not play a selective or dominant role in head and neck SCC. According to Jalouli et al., the EBV positivity rates in oral squamous cell carcinoma were found to be 80% in the United Kingdom, 71% in Sweden, and 70% in Norway. These values are significantly higher than the 10% rate found in our study. This difference may be attributed to our study cohort consisting of only 50 patients, which may limit statistical generalizability. HNC patients with newly diagnosed untreated tumors undergoing biopsy are a relatively difficult group to recruit in our region within a short study window [17].

As for CMV, existing studies tend to focus not on its direct oncogenic potential in HNCs but rather on its secondary roles in immunosuppression, tumor microenvironment modulation, and chemotherapy resistance [18]. Although some reports have linked CMV detection in tumor tissue to disease progression or poor prognosis, in our case series, CMV DNA positivity was low and similarly distributed across subtypes [19].

In the case of HSV, HSV DNA was detected in 3 patients. Abilaji et al. stated that HSV-1 and HSV-2 do not seem to act as direct carcinogens; the findings indicate that they may contribute to the development of HNC through immunological or virological pathways. Conducting stratified analyses based on tumor subsite and histology could reveal site-specific interactions, while further mechanistic studies are necessary to clarify how HSV influences immune responses or interacts with HPV oncogenes [20].

The detection of viral DNA predominantly in patients aged 50 years and older was noteworthy; however, age-based subgroup analysis did not reveal a statistically significant difference. Previous literature suggests that HPV-related HNCs are more frequent among younger adults, whereas classical risk factor-associated cases tend to occur at older ages [21]. Chaturvedi et al. reported an increasing trend of HPV-positive oropharyngeal cancers in individuals under 50, suggesting that viral factors may be more prominent in this subgroup [22]. In our study, however, HPV, EBV, and CMV DNA positivity in tissue samples was also observed in older individuals, supporting the hypothesis that latent viral infections may reactivate with age or that age-related immune changes could facilitate viral oncogenesis. Based on our data, CMV, HSV-1, and EBV DNA were detected in biopsy samples from patients with various HNCs, with HSV-1 predominantly detected in tongue SCC, EBV mainly in laryngeal and retromolar SCC, and CMV distributed across multiple sites. While CMV and EBV DNA positivity appeared sporadic and did not cluster within specific tumor localizations, HSV-1 showed a tendency to be associated with tongue SCC, suggesting a possible site-preferential presence. Conducting such stratified analyses, even when non-significant, may help clarify whether these viruses play differential roles across HNC subtypes.

Tissue-based DNA analysis showed higher positivity rates for CMV, EBV, and HPV. Serologically, high IgG positivity reflected past exposure, whereas low IgM rates indicated limited acute infections. Notably, in one patient, CMV IgG and CMV IgM were found to be positive simultaneously in a CMV DNA-positive biopsy sample, suggesting a concurrent or recently reactivated infection.

Serological IgG positivity was also higher in older age groups, indicating widespread past or persistent infections. These results are likely associated with immunological changes due to aging and cumulative lifetime exposure to these viruses [23]. It should be noted that the associations in this study are primarily based on viral DNA detected in tissue and blood, reflecting current or latent viral presence rather than prior exposure. Serological positivity (IgG/IgM) indicates past or recent exposure but does not confirm viral involvement in the tumor itself. While high IgG rates suggest widespread exposure among patients, these antibodies alone cannot establish a causal or local effect in oncogenesis. It remains possible that previous infections contributed to tumor development through transient infection or immune-mediated mechanisms, even in the absence of detectable viral DNA. Future studies integrating serology, tissue DNA, and viral RNA expression could help clarify the potential role of prior viral exposure in head and neck cancer pathogenesis. Serologically, high IgG positivity reflected past exposure, whereas low IgM rates indicated limited acute infections. The serological analysis was primarily performed to determine previous exposure rather than direct association with tumor development. These findings provide complementary insight into the cohort’s viral history, suggesting that latent or past infections may play a role even when DNA is undetectable in tissues.

Serological IgG positivity was higher in older age groups, reflecting widespread past or persistent infections [23]. Although HPV DNA positivity appeared higher in females (16.7%) than males (2.6%), this difference was not statistically significant (*p* = 0.14), and EBV and CMV DNA positivity was observed only in males without reaching significance, suggesting that gender-related patterns may be context-dependent rather than conclusive [24]. These differences may stem from geographic and cultural factors, as well as variations in risk behaviors, sexual practices, and biological immune responses. Our findings suggest that the impact of gender on HPV positivity may be context-dependent, and the potential risk of HPV-related HNC in women should not be overlooked. EBV and CMV DNA positivity were observed only in male patients, but the limited number of cases prevented statistical significance. The literature contains limited data on this matter, and gender differences are generally attributed to risk exposures and behavioral patterns.

In the study conducted by Polz-Gruszka and colleagues, HPV was detected in 32.5% of 80 patients with laryngeal and oropharyngeal carcinoma; HPV type 16 was detected in 22.5% of these patients; other types, 59, 45 and 68, were detected in four cases (10%). HPV was detected more frequently in laryngeal carcinoma (36%) than in oropharyngeal carcinoma (26.7%). However, this difference was not statistically significant. EBV was detected in 57.5% of the samples studied; 60% of these were laryngeal carcinomas and 53.3% were oropharyngeal carcinomas. CMV was detected in eight cases (10%). CMV was detected more frequently in laryngeal cancer than in oropharyngeal cancer. This difference was not statistically significant. Co-infection with one or more viruses was detected in 30% of cases. The most common co-infection was with EBV and HPV (15%). Among other viruses, co-infection rates were EBV and CMV 7.5%; HPV and CMV 5%; HPV, CMV and EBV were detected in one case. The frequency of HPV DNA increased with the age of the patients, being 8.3% in patients under 50 years of age and 83.3% in the group of patients over 70 years of age. EBV was most frequently detected in the under-50 age group, at a rate of 66.7%. CMV prevalence was highest in patients under 50 years of age, at 16.7% [25].

The study conducted by Naqvi and colleagues included 58 patients with oral squamous cell carcinoma (OSCC). The mean age of the patients was 42 years (standard deviation: ±12 years) and ranged from 26 to 70 years. Males accounted for 82%, while females accounted for 18%. All cases were negative for HPV. CMV was detected in only three cases (5.17%), while EBV was positive in 15 cases (25.86%). Of the 58 cases, 24 (41.37%) were localised in the buccal cavity, 17 (29.3%) in the tongue, 11 (18.96%) in the lips, and 6 (10.34%) in the palate. Among patients with OSCC localised in the buccal cavity, only 2 (8.33%) were CMV-positive and 10 (41.66%) were EBV-positive; none were HPV-positive. Of the 17 cases affecting the tongue, only 1 (5.55%) was positive for CMV and 4 (23.52%) were positive for EBV, while no HPV-positive cases were detected. In OSCC patients with lesions localised on the lips, no one tested positive for HPV and EBV, while only 1 (9.09%) tested positive for CMV. In the six cases with lesions localised on the palate, none of the patients tested positive for HPV, EBV, or CMV [26].

This study has several limitations, including a relatively small sample size and being a single-center study. In particular, the small number of cases in certain subgroups (e.g., rare tumors like retromolar SCC) limited statistical power and generalizability. Additionally, potential confounding variables such as immune status, treatment history, and tumor stage could not be comprehensively analyzed in this cohort. Nevertheless, the findings provide valuable and original insights into the presence and distribution of viral agents in HNCs.

## 5. Conclusions

This study provides new insights into the viral etiology of head and neck cancers (HNCs) by systematically assessing multiple viral agents at both molecular and serological levels in a regional cohort. Our findings demonstrate that HPV has a selective oncogenic role, particularly in oral cavity and tongue squamous cell carcinomas, while EBV, CMV, and HSV were detected at lower rates and showed no distinct distribution patterns. Importantly, this investigation reveals that combining DNA-based tissue analysis with serological profiling can uncover both active and past viral exposures, offering a more comprehensive understanding of viral involvement in HNC pathogenesis. Although demographic factors such as age and sex did not show statistically significant associations with viral positivity, the data suggest potential context-dependent variations in viral influence. By providing detailed prevalence data, genotype information, and the relationship between viral presence and tumor subtype, this study contributes novel evidence on the selective role of HPV and the limited involvement of other viruses, thereby expanding the current knowledge on viral contributions to head and neck carcinogenesis. These findings support targeted HPV screening and vaccination strategies in high-risk populations and highlight the need for larger, multicenter studies to further elucidate viral etiological mechanisms in HNCs.

## Figures and Tables

**Table 1:** Demographic characteristics of all cases.

Demographics	Value
Total Cases (n)	50
Mean Age (±SD)	57.9 (±13.0)
Age Scale (min.–max.)	24.0–86.0
Male (%)	38 (73.1%)
Female (%)	12 (23.1%)
Common Tumor Subtypes (%)	Laryngeal SCC * (34.6%), Tongue SCC * (21.2%), Retromolar SCC * (3.8%)
Smoking Status (%)	88% (n = 44/50)
Alcohol Use (%)	18% (n = 9/50)

* SCC, squamous cell carcinoma.

**Table 2:** CMV, EBV, HSV, and HPV DNA positivity in blood and tissue samples of patients with head and neck cancer.

Virus	Sample Type	Positive (n)	Positivity (%)	95% CI	*p*-Value
HPV	Blood	2	4.0%	0.5–13.7%	0.63
	Tissue Biopsy	3	6.0%	1.3–16.5%	
EBV	Blood	1	2.0%	0.1–10.5%	0.21
	Tissue Biopsy	5	10.0%	3.3–21.8%	
CMV	Blood	0	0.0%	0.0–7.1% *	0.12
	Tissue Biopsy	4	8.0%	2.2–19.2%	
HSV	Blood	0	0.0%	0.0–7.1% *	0.18
	Tissue Biopsy	3	6.0%	1.3–16.5%	

CMV, Cytomegalovirus, HSV, Herpes simplex virus, EBV, Epstein–Barr virus, HPV, Human papillomavirus. * *p*-values represent comparisons between blood and tissue positivity rates for each virus (Fisher’s Exact Test). *p* < 0.05 was considered statistically significant.

**Table 3:** IgM and IgG positivity rates of CMV, HSV-1/2, EBV, and HPV in patients with head and neck cancer.

Serological Parameter	Positive (n)	Positive (%)	*p*-Value (vs. DNA Positivity)
CMV IgM	3	6%	0.47
CMV IgG	47	94%	0.89
HSV 1 IgM	0	0.0%	-
HSV 1 IgG	49	98%	0.67
HSV 2 IgM	1	2%	0.72
HSV 2 IgG	15	30%	0.55
EBV IgM	1	2%	0.41
EBV IgG	47	94%	0.65
HPV IgM	4	8%	0.36
HPV IgG	3	6%	0.29

CMV, Cytomegalovirus, HSV, Herpes simplex virus, EBV, Epstein–Barr virus, HPV, Human papillomavirus. *p*-values indicate comparisons between serological antibody positivity and corresponding viral DNA detection (Fisher’s Exact Test).

## Data Availability

Data is contained within the article or Supplementary Materials.

## Supplementary Materials

The following supporting information can be downloaded at: https://www.mdpi.com/article/10.3390/biology14111523/s1, Age, Gender, Diagnose, CMV DNA (Blood), HSV DNA (Blood), EBV DNA (Blood), HPV DNA (Blood), HPV genotype (Blood), HPV DNA (Tissue), HPV genotype (tissue), CMV DNA (Tissue), HSV DNA (Tissue), EBV DNA (Tissue), CMV IgM, CMV IgG, HSV 1 IgM, HSV 1 IgG, HSV 2 IgM, HSV 2 IgG, EBV IgM, EBV IgG, HPV IgM, HPV IgG, Cigarette consumption (pack/year), Alcohol consumption, Have you had surgery in the dental/oral area before?

## Abbreviations

The following abbreviations are used in this manuscript:

SCC	Squamous cell carcinoma
PCR	Polymerase chain reaction
CMV	Cytomegalovirus
HSV	Herpes simplex virus
EBV	Epstein–Barr virus
HPV	Human papillomavirus
