# Supplementary material for: HPV, EBV, CMV, and HSV in Head and Neck Cancer: Molecular Detection, Seroprevalence, and Clinical Correlations
Source: Biology (Basel). 2025 Oct 30;14(11):1523. doi: 10.3390/biology14111523 (PMC12650686; doi:10.3390/biology14111523)
Supplement: Supplementary file 1 [file biology-14-01523-s001.zip › biology-3908247-supplementary.pdf]

No	Age	Gender	Diagnose	CMV DNA (Blood)	HSV DNA (Blood)	EBV DNA (Blood)	HPV DNA (Blood)	HPV genotype (Blood)	HPV DNA (Tissue)	HPV genotype (tissue	CMV DNA (Tissue)	HSV DNA (Tissue)	EBV DNA (Tissue)
1	54	F	SERT DAMAK SCC	negative	negative	negative	negative	Not detected	negative	Not detected	negative	negative	negative
2	56	M	DİL SCC	negative	negative	negative	negative	Not detected	negative	Not detected	negative	negative	negative
3	58	M	LARENKS SCC	negative	negative	negative	negative	Not detected	negative	Not detected	negative	negative	negative
4	53	F	DİL SCC	negative	negative	negative	negative	Not detected	negative	Not detected	negative	negative	negative
5	52	F	DİL SCC	negative	negative	negative	positive	18	negative	Not detected	negative	negative	negative
6	61	M	LARENKS SCC	negative	negative	negative	negative	Not detected	negative	Not detected	negative	negative	negative
7	72	M	MAXİLLA SCC	negative	negative	negative	negative	Not detected	negative	Not detected	negative	negative	negative
8	60	M	MAXİLLA ACC	negative	negative	negative	negative	Not detected	negative	Not detected	negative	negative	negative
9	43	M	AĞIZ TABANI SCC	negative	negative	negative	negative	Not detected	negative	Not detected	negative	negative	negative
10	44	F	DİL SCC	negative	negative	negative	negative	Not detected	positive	45	negative	HSV 1 positive	negative
11	25	F	DİL SCC	negative	negative	negative	negative	Not detected	negative	Not detected	negative	negative	negative
12	53	M	LARENKS SCC	negative	negative	negative	negative	Not detected	negative	Not detected	negative	negative	negative
13	86	M	LARENKS SCC	negative	negative	negative	negative	Not detected	negative	Not detected	negative	negative	negative
14	48	M	LARENKS SCC	negative	negative	negative	negative	Not detected	negative	Not detected	negative	negative	negative
15	40	M	LARENKS SCC	negative	negative	negative	negative	Not detected	negative	Not detected	negative	negative	negative
16	49	F	DİLDE ACC	negative	negative	negative	negative	Not detected	negative	Not detected	negative	negative	negative
17	68	M	LARENKS SCC	negative	negative	negative	negative	Not detected	negative	Not detected	negative	negative	negative
18	43	F	DİL SCC	negative	negative	negative	negative	Not detected	negative	Not detected	negative	negative	negative
19	70	M	LARENKS SCC	negative	negative	negative	negative	Not detected	negative	Not detected	negative	negative	negative
20	68	M	DİL SCC	negative	negative	negative	negative	Not detected	negative	Not detected	negative	negative	negative
21	81	F	MANDİBULA SCC	negative	negative	negative	negative	Not detected	negative	Not detected	negative	negative	negative
22	65	M	LARENKS SCC	negative	negative	negative	negative	Not detected	negative	Not detected	positive	negative	positive
23	59	M	LARENKS SCC	negative	negative	negative	negative	Not detected	negative	Not detected	negative	negative	negative
24	54	F	NAZAL RHABDOMYOSARKOM	negative	negative	negative	negative	Not detected	negative	Not detected	negative	negative	negative
25	24	M	OLFAKTÖR NÖROBLASTOM	negative	negative	negative	negative	Not detected	negative	Not detected	negative	negative	negative
26	50	M	LARENKS SCC	negative	negative	negative	negative	Not detected	negative	Not detected	negative	negative	negative
27	51	M	RETROMOLAR SCC	negative	negative	negative	positive	Not detected	positive	18	negative	negative	positive
28	62	F	DİL SCC	negative	negative	negative	negative	Not detected	negative	Not detected	negative	negative	negative
29	49	F	MANDİBULA OSTEOSARKOM	negative	negative	negative	negative	Not detected	negative	Not detected	negative	negative	negative
30	64	M	RETROMOLAR SCC	negative	negative	negative	negative	Not detected	negative	Not detected	negative	negative	negative
31	69	M	NAZAL VESTİBÜL SCC	negative	negative	negative	negative	Not detected	negative	Not detected	positive	negative	negative
32	41	M	MAXİLLA SCC	negative	negative	negative	negative	Not detected	negative	Not detected	negative	negative	negative
33	42	M	NAZAL KAVİTE SCC	negative	negative	negative	negative	Not detected	negative	Not detected	negative	HSV 1 positive	negative
34	80	M	DİL SCC	negative	negative	negative	negative	Not detected	negative	Not detected	positive	negative	negative
35	60	M	RETROMOLAR TRİGON SCC	negative	negative	negative	negative	Not detected	negative	Not detected	positive	negative	positive
36	62	M	DİL SCC	negative	negative	negative	negative	Not detected	negative	Not detected	negative	HSV 1 positive	negative
37	61	M	LARENKS SCC	negative	negative	negative	negative	Not detected	negative	Not detected	negative	negative	negative
38	77	M	LARENKS SCC	negative	negative	negative	negative	Not detected	negative	Not detected	negative	negative	negative
39	51	M	NAZOFARENKS SCC	negative	negative	negative	negative	Not detected	negative	Not detected	negative	negative	negative
40	69	M	LARENKS SCC	negative	negative	negative	negative	Not detected	negative	Not detected	negative	negative	negative
41	65	M	LARENKS SCC	negative	negative	negative	negative	Not detected	negative	Not detected	negative	negative	negative
42	70	M	PAROTİS GLAND SCC	negative	negative	negative	negative	Not detected	negative	Not detected	negative	negative	negative
43	50	M	İĞSİ HÜCRELİ SARKOM	negative	negative	negative	negative	Not detected	negative	Not detected	negative	negative	negative
44	49	M	OTİS-MALİGN EPİTELYAL KARSİ	negative	negative	positive	negative	Not detected	negative	Not detected	negative	negative	positive
45	74	M	LARENKS SCC	negative	negative	negative	negative	Not detected	negative	Not detected	negative	negative	positive
46	71	M	SİNONAZAL SCC	negative	negative	negative	negative	Not detected	negative	Not detected	negative	negative	negative
47	53	M	LARENKS SCC	negative	negative	negative	negative	Not detected	negative	Not detected	negative	negative	negative
48	68	F	DİL SCC	negative	negative	negative	negative	Not detected	positive	69	negative	negative	negative
49	60	M	LARENKS SCC	negative	negative	negative	negative	Not detected	negative	Not detected	negative	negative	negative
50	60	M	BUKKAL MUKOZA SCC	negative	negative	negative	negative	Not detected	negative	Not detected	negative	negative	negative

No	CMV IgM	CMV IgG	HSV 1 IgM	HSV 1 IgG	HSV 2 IgM	HSV 2 IgG	EBV IgM	EBV IgG	HPV IgM	HPV IgG	CIGARETTE PACK/YEAR	Alcohol consumption
1	negative	positive	negative	positive	negative	positive	negative	positive	negative	negative	0	
2	negative	positive	negative	positive	negative	positive	negative	positive	negative	negative	50	Günde 3 kadeh
3	negative	positive	negative	positive	negative	positive	negative	positive	negative	negative	40	0
4	positive	positive	negative	positive	negative	negative	negative	positive	negative	negative	5	0
5	negative	positive	negative	positive	negative	negative	negative	positive	negative	negative	0	0
6	negative	positive	negative	positive	negative	negative	negative	positive	negative	negative	0	0
7	negative	positive	negative	positive	negative	negative	negative	positive	negative	negative	0	0
8	negative	positive	negative	positive	negative	positive	negative	positive	negative	negative	0	0
9	negative	positive	negative	positive	negative	negative	negative	negative	negative	negative	30	0
10	negative	positive	negative	positive	negative	negative	positive	positive	negative	negative	15	0
11	negative	positive	negative	positive	negative	negative	negative	positive	positive	negative	0	0
12	negative	positive	negative	positive	negative	positive	negative	positive	positive	positive	30	0
13	negative	positive	negative	positive	negative	negative	negative	positive	negative	negative	50	0
14	negative	positive	negative	positive	negative	negative	negative	positive	negative	negative	30	0
15	negative	positive	negative	positive	negative	negative	negative	positive	negative	negative	50	Günde 2 kadeh
16	negative	positive	negative	positive	negative	negative	negative	positive	negative	negative	2	0
17	negative	positive	negative	positive	negative	positive	negative	positive	negative	negative	68	0
18	negative	positive	negative	positive	negative	negative	negative	positive	negative	negative	43	0
19	negative	positive	negative	positive	negative	negative	negative	positive	negative	negative	70	0
20	negative	positive	negative	positive	negative	positive	negative	positive	negative	negative	90	0
21	negative	positive	negative	positive	negative	negative	negative	positive	negative	negative	81	0
22	positive	positive	negative	positive	negative	negative	negative	positive	negative	negative	90	0
23	negative	positive	negative	positive	negative	positive	negative	positive	negative	negative	70	Günde 4 kadeh
24	negative	positive	negative	positive	negative	negative	negative	positive	negative	negative	54	0
25	negative	positive	negative	negative	negative	negative	negative	negative	negative	negative	23	0
26	negative	positive	negative	positive	negative	negative	negative	positive	negative	negative	49	Sosyal içici
27	negative	positive	negative	positive	negative	negative	negative	positive	negative	negative	51	Günde 2 kadeh
28	negative	positive	negative	positive	negative	negative	negative	positive	negative	negative	62	0
29	negative	positive	negative	positive	negative	positive	negative	negative	negative	negative	49	0
30	negative	positive	negative	positive	negative	positive	negative	positive	negative	negative	64	0
31	negative	positive	negative	positive	negative	negative	negative	positive	negative	negative	45	0
32	negative	negative	negative	positive	negative	negative	negative	positive	negative	negative	9	0
33	negative	positive	negative	positive	negative	negative	negative	positive	negative	negative	4	0
34	ŞÜPHELİ	negative	negative	positive	negative	negative	negative	positive	negative	negative	10	0
35	negative	positive	negative	positive	negative	negative	negative	positive	negative	negative	40	Sosyal içici
36	positive	positive	negative	positive	negative	positive	negative	positive	negative	negative	2	0
37	negative	positive	negative	positive	negative	negative	negative	positive	negative	negative	25	0
38	negative	positive	negative	positive	negative	negative	negative	positive	negative	negative	40	5 günde 1 kadeh
39	negative	positive	negative	positive	negative	negative	negative	positive	positive	positive	7	0
40	negative	positive	negative	positive	negative	negative	negative	positive	negative	negative	40	0
41	negative	positive	negative	positive	negative	positive	negative	positive	negative	negative	15	0
42	negative	positive	negative	positive	negative	positive	negative	positive	negative	negative	40	0
43	negative	positive	negative	positive	negative	negative	negative	positive	negative	negative	25	0
44	negative	positive	negative	positive	negative	negative	negative	positive	negative	negative	25	0
45	negative	positive	negative	positive	negative	negative	negative	positive	negative	negative	75	0
46	negative	positive	negative	positive	negative	negative	negative	positive	positive	positive	35	0
47	negative	positive	negative	positive	positive	negative	negative	positive	negative	negative	30	Günde 2 kadeh
48	negative	positive	negative	positive	negative	positive	negative	positive	negative	negative	5	0
49	negative	negative	negative	positive	negative	positive	negative	positive	negative	negative	40	Sosyal içici
50	negative	positive	negative	positive	negative	negative	negative	positive	negative	negative	10	0

Have you had surgery in the dental/oral area before?

-
-
-
-
-
-

Sağ üst çenede 2 adet ve sol alt çenede 1 adet implant

si ile ilişkili kitle görülmesi üzerine diş çekimi ve kitle eksizyonu ameliyatı yapılan hasta

-
-
-
-
-
-
-
-
-
-
-
-
-
-
-
-
-
-
-
-

Diş hekimliği biyopsi

08 yılında sol bukkal mukoza liken planus nedeniyle dış merkezde operasyon

-
-
-
-
-

2007 Sol parsiyel glossektomi+Sol BD

-
-
-
-
-
-
-
-
-
-
-

lunan hemanjiom nedeniyle birçok kez kriyoterapi ve intralezyoner enjeksiyon tedavisi öyküsü

-
-
